# Molecular analysis of the factorless internal ribosome entry site in *Cricket Paralysis virus* infection

**DOI:** 10.1038/srep37319

**Published:** 2016-11-17

**Authors:** Craig H. Kerr, Zi Wang Ma, Christopher J. Jang, Sunnie R. Thompson, Eric Jan

**Affiliations:** 1 Department of Biochemistry and Molecular Biology, University of British Columbia, Vancouver BC, Canada; 2 Department of Microbiology, University of Alabama at Birmingham, Birmingham, Alabama, USA

## Abstract

The dicistrovirus *Cricket Paralysis virus* contains a unique dicistronic RNA genome arrangement, encoding two main open reading frames that are driven by distinct internal ribosome entry sites (IRES). The intergenic region (IGR) IRES adopts an unusual structure that directly recruits the ribosome and drives translation of viral structural proteins in a factor-independent manner. While structural, biochemical, and biophysical approaches have provided mechanistic details into IGR IRES translation, these studies have been limited to *in vitro* systems and little is known about the behavior of these IRESs during infection. Here, we examined the role of previously characterized IGR IRES mutations on viral yield and translation in CrPV-infected Drosophila S2 cells. Using a recently generated infectious CrPV clone, introduction of a subset of mutations that are known to disrupt IRES activity failed to produce virus, demonstrating the physiological relevance of specific structural elements within the IRES for virus infection. However, a subset of mutations still led to virus production, thus revealing the key IRES-ribosome interactions for IGR IRES translation in infected cells, which highlights the importance of examining IRES activity in its physiological context. This is the first study to examine IGR IRES translation in its native context during virus infection.

Canonical eukaryotic translation initiation is a highly orchestrated series of steps involving 40S recruitment to the 5′ cap, scanning, 80S assembly and initiation at an AUG codon^1^. Twelve core translation factors are required to mediate cap-dependent translation^1^. All viruses utilize the host translation machinery for viral protein synthesis and some have evolved ingenious strategies to commandeer host ribosomes for their own benefit. Internal ribosome entry sites (IRESs) are one of the most well studied translation initiation mechanisms employed by some viruses to facilitate expression of their genomes. In general, IRESs are structured RNAs that directly recruit the ribosome using a subset of translation factors, thus providing an advantage during infection when cap-dependent translation is compromised^2^,^3^. IRESs are classified based on nucleotide and structural conservation as well as mechanism of translation initiation. The picornavirus IRESs require most of the canonical translation initiation factors and IRES-trans-acting factors whereas the hepatitis C virus-like IRESs are streamlined requiring only eIF2 and eIF3 to recruit the ribosome and initiate translation^4^,^5^. Extensive biochemical studies and the identification of factors required for IRES translation have yielded detailed insights into IRES mechanisms. The challenge is to identify the key steps and factors in IRES translation that impact virus infection.

One of the most well-studied and perhaps simplest IRES to date is within the intergenic region of the *Dicistroviridae*. Dicistroviruses are single-stranded positive sense RNA viruses with genomes sizes ranging from 8–10 kb that infect arthropods^6^.The namesake of these viruses stems from their unique dicistronic genome arrangement where each open reading frame (ORF) is driven by a distinct IRES which allows differential and temporal regulation of each ORF during infection^7^,^8^. The 5′ untranslated region (5′UTR) IRES directs translation of ORF1, which encodes the viral non-structural proteins, such as the RNA-dependent RNA polymerase and 3C protease (Fig. 1). Whereas the aforementioned intergenic region IRES (IGR IRES) facilitates translation of the viral structural proteins encoded in the second ORF (ORF2)^6^.

Through a unique mechanism, the IGR IRES binds directly to 40S and 80S ribosomes without the assistance of initiation factors or initiator Met-tRNA_i_ and initiates translation from a non-AUG codon^9^,^10^. Several structural and *in vitro* biochemical studies have revealed that the CrPV IGR IRES adopts a structure comprising of three pseudoknots (termed PKI, II, and III) and two stem-loops (SLIV and SLV)^11^,^12^,^13^,^14^,^15^,^16^,^17^,^18^,^19^. Together PKII and PKIII form the ribosome-binding domain, which mediates 40S and 80S recruitment, and the PKI domain functionally mimics the anticodon stem of a tRNA, which permits the IGR IRES to first occupy the ribosomal A site^12^,^13^,^15^,^16^,^18^,^19^,^20^. Upon initial occupancy of the A site by PKI, eukaryotic elongation factor 2 (eEF2) facilitates a pseudo-translocation step that involves movement of the PKI domain to the P site, leaving the A site clear for delivery of the first aminoacyl-tRNA by eEF1A^13^,^21^,^22^. The IRES undergoes a second eEF2-mediated translocation event without peptide bond formation; elongation then proceeds after^21^,^22^,^23^,^24^,^25^. The tRNA-like anticodon domain of the PKI domain sets the reading frame for IRES translation as it translocates through the decoding center of the ribosome^15^,^26^,^27^. The IGR IRES makes specific contacts with both ribosomal subunits. Stem-loops SLIV and SLV interact with uS7 and eS25 of the 40S subunit. Biochemical and structural data suggest that that uS7 and eS25 interact with and bridge both SLV and SLIV, thus these ribosomal proteins may have redundant yet crucial roles in binding to and positioning of the IGR IRES relative to the 40S subunit^13^,^14^,^28^,^29^. In support of these interactions, IGR IRES translation is abrogated in yeast lacking eS25^29^. The conserved L1.1 loop of the IGR IRES interacts with the L1 stalk of the 60S ribosomal subunit, which is reminiscent of interactions of the L1 stalk with an E-site deacylated tRNA^12^,^30^. The variable loop region (VLR) in the PKI domain, which is reported to facilitate ribosome positioning and eEF2-mediated pseudotranslocation, interacts with the β-hairpin loop of uS7 and helix 23 of 18S rRNA in the E site, suggesting that the VLR has a stabilizing role as the IRES translocates through the ribosome^25^,^31^,^32^. In addition to the ribosome, the IGR IRES may interact with eEF2 to facilitate its movement through the ribosome^31^. IGR IRES recruitment of the ribosome is inhibited by depleting pseudouridylation of rRNA, suggesting that specific rRNA modifications can affect IRES function^33^. Altogether, the contacts with the 80S ribosome allow the IGR IRES to operate as a highly tuned RNA element that can initiate translation in an unprecedented manner.

To date, studies on the *Dicistroviridae* IGR IRES have been limited to *in vitro* translation assays, reporter assays in tissue culture cells and orthologous systems (e.g. *S. cerevisiae,* cell extracts etc.). In this study, we use a recently developed infectious clone of CrPV, termed CrPV-3, which now allows the use of reverse genetics, to probe the IGR IRES mechanism in its native context^34^. Specifically, the physiological significance of well-established mutations in the IGR IRES is examined in the context of the entire CrPV genome and in CrPV-infected *Drosophila* cells.

## Results and Discussion

**Figure 1:**
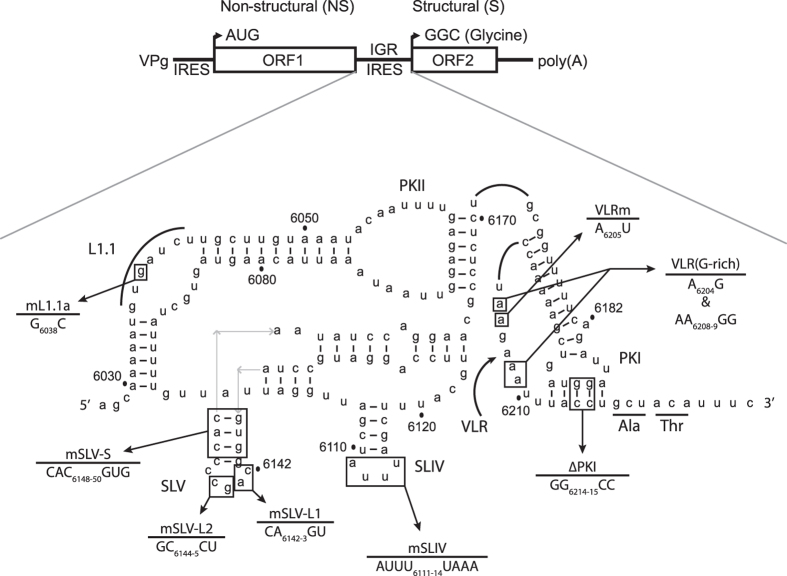
Secondary structure schematic of the CrPV Intergenic region IRES. Pseudoknots (PK) I, II, and III as well as stem-loops (SL) IV and V are indicated. The CCU triplet initially occupies the A site of the 80S ribosome and moves to the P site; translation then initiates at the GCU codon. Numbering refers to the nucleotide position within the CrPV RNA genome. Black dashes represent helical regions within the IRES and underlined residues represent the first two amino acids of the viral capsid protein. Dashed grey lines indicate regions that interact with the ribosome. Mutations generated in this study are depicted.

**Figure 2:**
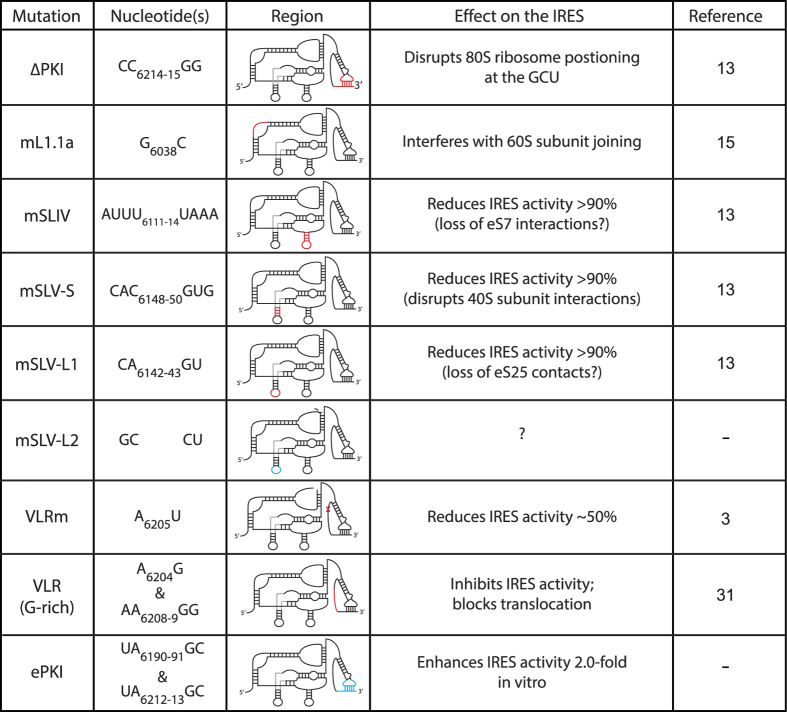
List of mutations generated in the CrPV IRES. Analysis of mutations within IGR IRES of CrPV-3 and their effects. Red indicates detrimental mutations while blue indicates enhancing or no effect mutations.

To assess the relevance of IGR IRES translation in CrPV infection, we systematically introduced a panel of known mutations that affect specific properties of IGR IRES translation within the CrPV-3 infectious clone (Figs 1 and 2). We generated a series of mutations as follows: (i) nucleotides CC_6214-15_ to GG (CC_6214-15_GG), which disrupts PKI base-pairing and effectively inhibits proper 80S positioning on the IRES^18^. (ii) Nucleotides within the loop of SLIV. The SLIV loop (nucleotides AUUU_6111-14_) makes contacts with ribosomal protein uS7 with the head of the 40S subunit. Mutating AUUU_6111-14_UAAA (mSLIV) markedly reduces translational activity and is reported to reduce 40S binding^13^,^18^,^25^. (iii) Nucleotides within the loop or helical stem of SLV were altered. Mutating the loop of SLV (mSLV-L1; CA_6142-43_GU) or disrupting the helical stem of SLV (mSLV-S; CAC_6148-50_GUG]) reduce translational activity of the IGR IRES, presumably due to loss of interactions with ribosomal protein eS25 (and potentially uS7), however these mutations on their own largely do not affect 40S binding^13^,^14^,^18^,^28^,^29^. We also made another mutation (mSLV-L2; GC_6144-45_CU) within the SLV loop to determine effects on nucleotide identity in this region. (iv) A single point mutation in the L1.1a loop of the IGR IRES (G_6038_C; mL1.1a). The G_6038_C mutation disrupts 60S but not 40S binding^30^. The L1.1a domain interacts with the L1 stalk, resembling contacts of an E-site tRNA^12^,^30^. (v) Two sets of mutations within the Variable Loop Region (VLR), A6205 to U (mVLR) and A6205G & AA6208-9GG (VLR(G-rich)), both of which reduce IRES translation^31^,^35^. The mVLR inhibits ribosome positioning whereas the VLR(G-rich) mutation has been shown to inhibit IRES-mediated translocation^31^,^35^. (vi) Mutations that are predicted to strengthen specific helical regions of the IGR IRES (UA_6190-91_GC/UA_6212-13_GC within PKI (ePKI)) and enhance IRES activity by ~2 fold using a bicistronic reporter assay in rabbit reticulocyte lysate (RRL) (unpublished, Chris Jang).

**Figure 3:**
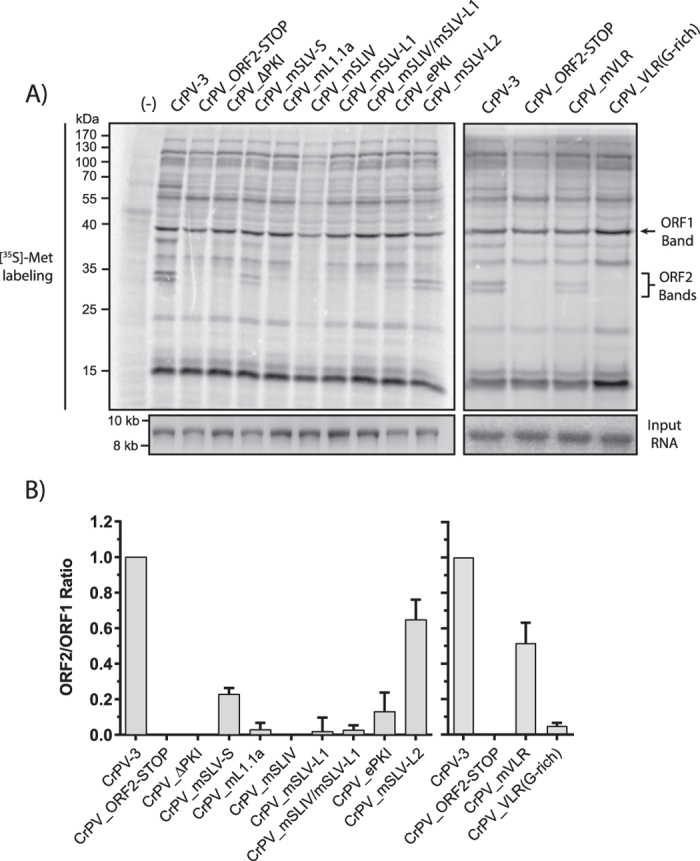
IGR IRES mutants are able to synthesize viral structural proteins in the context of the CrPV-3 genome. **(A)** *in vitro* synthesized RNA (2 μg) derived from CrPV-3 or the indicated mutant was incubated in Sf-21 extracts for 2 h at 30 °C in the presence of [^35^S]-methionine/cysteine. Extracts were resolved via SDS-PAGE and radioactive proteins were analyzed by phosphoimager analysis. A mutant containing a STOP codon in ORF2 of CrPV-2 (lane 3) was used as a control as it does not express structural proteins (see Materials and Methods)^34^. Shown is a representative gel from 3 independent experiments. 3 different batches of *in vitro* transcribed RNA were used. *bottom:* Agarose gel of *in vitro* transcribed input RNA. Note that uncropped images are shown in Supplementary Figure S1. A no RNA control is indicated by (−). Arrows indicate quantified bands. **(B)** Quantification of structural (ORF2) versus non-structural (ORF1) protein synthesis in Sf-21 extracts. Gels were quantified using ImageQuant. Bands used for quantitation are indicated with arrows in the gel above. Error bars represent the standard deviation.

Most structure/function analysis on the IGR IRES was performed using bicistronic reporter constructs in orthologous systems (e.g. RRL, human and yeast ribosomes)^36^,^37^,^38^. To determine IGR IRES activity in its native context, we monitored viral protein synthesis of *in vitro* transcribed CrPV-3 RNA incubated in insect Sf-21 translation extracts. We chose to utilize Sf-21 extracts for two main reasons: 1) these extracts are from the insect order *Lepidoptera* which have be shown to be previously susceptible to CrPV infection^6^,^39^ and 2) since the IGR IRES interacts with the conserved core of the ribosome, mechanisms observed between *Lepitdoteran* or *Dipteran* ribosomes are likely similar. Incubation of CrPV-3 RNA in the presence of [^35^S]-cysteine/methionine resulted in the synthesis of viral proteins ranging from ≤15 kDa to ≥170 kDa, similar to what was observed previously^34^. The amount of translation was quantified by comparing the intensity of ORF2 protein synthesized to ORF1. Since ORF1 is translated independently from ORF2, the level of ORF1 translation can be used as a baseline for translation from the viral RNA. Placement of a stop codon downstream of the IGR IRES completely abolished structural protein synthesis (Fig. 3)^34^. In agreement with *in vitro* studies, no structural protein synthesis was detected in mutant CrPV-3 ∆PKI, mL1.1a, mSLV-L1, mSLIV, VLR(G-rich), and a double mutant mSLIV/mSLV-L1 that contains mutations in both SLIV and SLV loops (Fig. 3). mSLV-L2, mSLV-S and mVLR reduced protein synthesis by approximately 40–80% (Fig. 3) In the case of mSLV-S, this is in agreement with previous *in vitro* data using compensatory mutations that the structure of SLV is integral to IRES function^18^. For VLRm, this data suggests that the nucleotide identity of the VLR is important for IRES function as seen previously *in vitro*^31^. Furthermore, specific mutations within the SLV loop (mSLV-L1 vs mSLV-L2) can affect IRES translation distinctly suggesting the nucleotide identity of the loop region may be important to IRES function. Mutant ePKI, which is reported to enhance IGR IRES activity in a bicistronic assay in RRL, resulted in an ~90% decrease in structural proteins synthesis compared to WT CrPV-3. This result demonstrates that the context of the IRES and/or the type of translation extract system can influence IRES activity. In general, mutations within the IGR IRES inhibited ORF2 structural protein synthesis in Sf-21 extracts in the CrPV-3 infectious clone.

**Figure 4:**
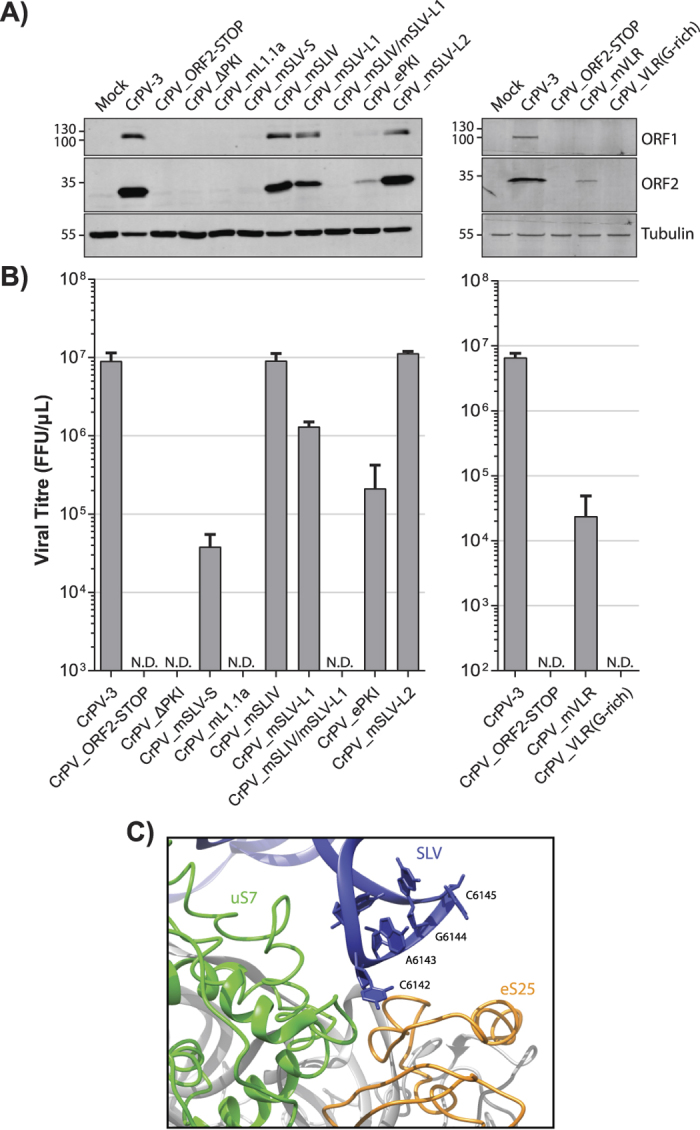
Viruses harboring mutations in the IGR IRES produce viable virus. **(A)** Western blot analysis of viral 3CD (ORF1) and VP2 (ORF2) from lysates of cells transfected with the indicated CrPV genomic RNA at 48 h.p.t. Note that uncropped images are shown in Supplementary Figure S2 **(B)** Titres of transfected CrPV IGR IRES mutants. 2.5 × 10^6^ S2 cells were transfected with 3 μg of the indicated *in vitro* transcribed genomic RNA. Titres were measured from the cell pellet as described in the Materials and Methods at 48 h.p.t. Shown are averages from at least 3 independent experiments (±S.D.). N.D. = Not detected. **(C)** Zoomed in segment from a cryo-EM structure of the 80S ribosome from *Kluveromyces lactis* bound to the CrPV IGR IRES (PDB ID 4V92) (Republished from Cell14 licensed under CC by 3.0). SLV of the IRES is coloured in blue while ribosomal protein eS25 and uS7 are coloured in orange and green, respectively.

We next investigated whether these IGR IRES mutations had an effect on viral infectivity. We transfected equal amounts of *in vitro* transcribed wild type or mutant CrPV-3 RNA into S2 cells and monitored cytopathic effects, viral protein expression and viral titres. Transfection of *in vitro* synthesized CrPV-3 RNA yielded cytopathic effects (CPE) including membrane blebbing, cell clumping, and lysis as seen previously (data not shown)^34^. Moreover, RdRP (ORF1) and VP2 (ORF2) proteins were readily detected by immunoblotting and virus production was observed 48 hours post transfection (Fig. 4A,B). As shown previously, introduction of a stop codon downstream of the IGR IRES did not lead to viral protein synthesis or virus production (ORF2-STOP)^34^. Similarly, no viral proteins or viral titre were detected in S2 cells transfected with mutant CrPV-3 ∆PKI, mL1.1a, VLR(G-rich), or double mutant mSLIV/mSLV-L (Fig. 4A,B). However, transfection of mutant CrPV-3 mSLV-S, mSLV-L, mSLIV, ePKI, eSLV and mVLR resulted in viral protein expression and yielded virus production to varying extents. Compared to wild-type CrPV-3, viral yield was lower for cells transfected with mutant CrPV-3 mSLV-S, mSLV-L, ePKI and mVLR (Fig. 4A,B). In the case of ePKI, it is possible that increasing the rigidity of PKI by strengthening the base-pairing is not favorable in this context as the IGR IRES may require innate flexibility in the tRNA-mimicry region to facilitate IRES translocation. For some mutations such as ΔPKI, mL1.1a, and VLR(G-rich), the loss of IRES translation activity correlated with the lack of virus production, thus demonstrating the importance of these domains in directing IRES translation during virus infection. Surprisingly, several mutations such as mSLIV, mSLV-S, and mSLV-L1 yielded robust virus production yet were IRES-translation compromised. For instance, while the AUUU_6111-14_ loop sequence of SLIV is absolutely conserved throughout dicistroviruses, strongly suggesting nucleotide context importance, mutating AUUU to UAAA still yielded similar viral titres as the wild-type CrPV-3. By contrast, mutant mVLR, which decreased translation *in vitro* by only 50% (Fig. 3), resulted in a 2-log decrease in viral yield (Fig. 4). It is possible that *in vitro* assays are not sensitive enough or only capture specific properties of these elements on IRES translation and that the full potential are only observed under more physiological conditions. For example, these RNA elements may sample more structural conformations under virus infection that allow viral protein synthesis to occur. Alternatively, there may be functional redundancy in these elements. Although structural studies indicate that SLIV and SLV interact with distinct regions of the 40S subunit, uS7 and eS25, biochemical data suggest that eS25 makes contacts with both loop regions of SLV and SLIV, which may explain how mutations in either stem-loop alone are not sufficient to abolish viral translation in infected cells, yet when combined can prevent viral protein synthesis and virus production (Fig. 4B; see mSLIV/mSLV-L)^14^,^28^.

Interestingly, both SLV loops mutants, SLV-L1 and SLV-L2, yielded different results. Mutating nucleotides CA_6142-43_ decreased overall viral yield and ORF2 production while mutating nucleotides GC_6144-45_ did not have a significant affect despite being within the same vicinity (Fig. 4A,B; compare mSLV-L1 and mSLV-L2). Cryo-EM structural data of the CrPV IGR IRES bound to the 80S ribosome of *Kluyveromyces lactis*, indicate that nucleotide C_6142_ of SLV is in a flipped out orientation that is in close proximity to ribosomal protein uS7, while nucleotides GC_6144-45_ face inward into the loop (Fig. 4C)^13^,^17^. The position and conformation of C_6142_ when bound to the ribosome could explain the discrepancies observed between a detrimental mutation (mSLV-L1) and a mutation with no apparent affect (mSLV-L2). Alternatively, mutating C_6142_ to G may potentially lead to an additional base pair with C6146 in SLV, resulting in a lengthening of the helical stem and a reduction of the loop to 3 nucleotides, which may be insufficient for eS25 binding. At any rate, the nucleotide identity of SLV appears to be crucial for IRES function.

Mutations within the VLR have been shown to affect ribosome positioning and translocation on the IRES^31^,^32^. Recent cryo-EM studies reveal that the dynamic and flexible VLR becomes ordered upon the PKI translocation from the A to P site, resembling a post-translocated ribosome^17^,^25^. At this state, the VLR interacts with R148 and R157 in β-hairpin of uS7, which resembles an E-site tRNA anticodon stem loop interaction^25^. We show that mutations in VLR that likely disrupt these key ribosome-IRES interactions had a significant inhibitory effect on virus production (Fig. 4), thus further supporting the importance of this domain in directing viral structural protein synthesis during infection.

**Figure 5:**
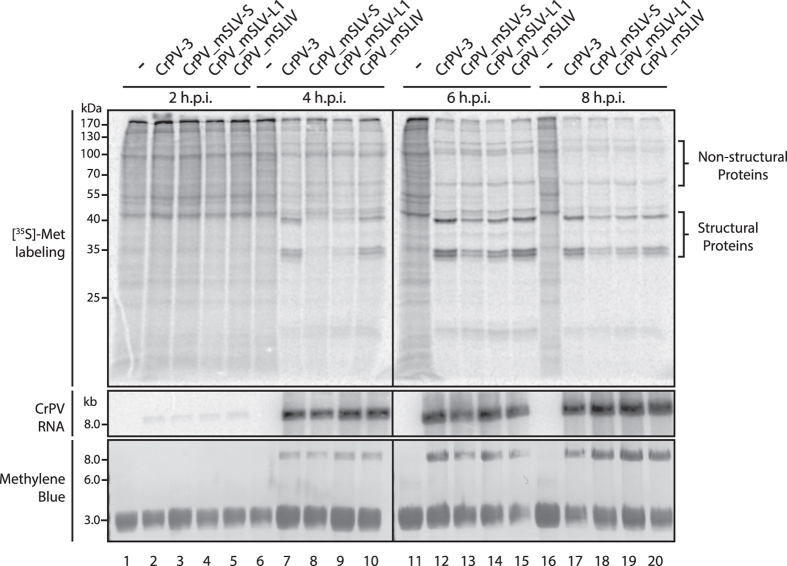
Viable viruses with mutated IGR IRESs are impaired in viral growth at the translational level. S2 cells were mock-infected (−) or infected with CrPV-3 or the indicated mutant (MOI 10). Cells were metabolically labeled with [^35^S]-methioine/cysteine for the last 30 minutes of infection. Lysates were subjected to SDS-PAGE and Northern blot analysis. Accumulation of viral RNA was monitored by probing for the CrPV RNA genome. Note that uncropped images are shown in Supplementary Figure S3.

Since mutant CrPV-3 mSLV-S, mSLV-L1 and mSLIV resulted in a productive infection (Fig. 4), we next investigated whether the observed reduction in viral loads was due to diminished viral translational activity or replication. To address this, we infected S2 cells with wild type CrPV-3 or mutant virus and monitored viral protein synthesis via [^35^S]-met/cys metabolic pulse-labeling and viral RNA by Northern blot analysis (Fig. 5). There was no observable difference in accumulation of viral RNA between wild type or any of the mutant viruses suggesting that RNA replication is unaffected (Fig. 5). However, as early as 4 hours post-infection there is a noticeable reduction in viral structural protein synthesis when a mutation is present either in the loop (mSLV-L1) or stem (mSLV-S) of SLV with the latter being more detrimental, while no reduction is observed with a SLIV mutation (Fig. 5). These data corroborated viral titres observed after RNA transfection (Fig. 4A) and altogether indicate that IGR IRES-dependent translation is an essential step in promoting virus infection.

Structural and biochemical studies on the dicistrovirus IGR IRES has yielded enormous insights into the detailed mechanism of IRES-mediated translation, ribosome dynamics and decoding. However, all of these studies are *in vitro* using reconstituted systems and translation extracts and thus, the context of IGR IRES translation in a virus system was lacking. Here, we now complement these studies by providing a physiological context of IGR IRES translation in a virus infection system, thus emphasizing the importance of specific structural elements of the IRES in its native context. The use of the CrPV infectious clone provides a powerful biological framework for pinpointing the relevant IGR IRES mechanistic details in a physiological virus system.

## Materials and Methods

### Cell culture and virus

*Drosophila* Schneider line 2 (S2) cells were maintained and passaged in Shield’s and Sang medium (Sigma) supplemented with 10% fetal bovine serum. Determination of CrPV viral titres and yield were performed as previously described^40^. Briefly, a total of 1.5 × 10^6^ S2 cells we incubated with serial dilutions of virus for 30 min, then resuspended in media, plated into a 96-well plate coated with concavilin A (0.5 mg/mL; Calbiochem) and incubated at 25 °C for 8 h. Cells were then washed with PBS before being fixed with 3% paraformaldehyde for 15 min followed by methanol for 10 min. The fixed cells were then incubated with an anti-ORF2 antibody (1:250 dilution in 5% bovine serum albumin in PBS) for 1 h at room temperature. Subsequently, cells were washed three times with PBS and incubated with a Texas Red IgG anti-rabbit (1:500 dilution in 5% bovine serum albumin in PBS; Invitrogen) for 1 h at room temperature. Finally, cells were washed with PBS stained with Hoechst dye (0.5 μg/mL). The amount of infected cells was quantified after plates were analyzed with a Cellomics Arrayscan HCS instrument. Through serial dilutions of CrPV, the FFU/mL can be calculated. Each titre is the result of at least three replicate experiments.

CrPV-3 and mutant viruses were generated from *Drosophila* S2 cells using an adapted protocol^41^. Briefly, 5.0 × 10^7^ S2 cells were transfected with *in vitro* transcribed RNA derived from pCrPV-3 or mutant plasmids and incubated for 48 h. Cells were dislodged into the media, treated with 0.5% Igepal CA-630 (Nonidet P-40) and 0.1% 2-mercaptoethanol, and incubated on ice for 10 min. Cell debris was cleared by centrifugation at 13,800 RCF for 15 min at 4 °C. Viral particles were then concentrated by ultracentrifugation at 141,000 RCF for 2.5 h at 4 °C. The pellet was resuspended in PBS and sterilized through a 0.2 μM filter. All viruses were sequence verified via RT-PCR with primers directed against the CrPV IGR IRES.

### Construction of CrPV-3 mutants

Deleterious mutations were introduced into the pCrPV-3 clone at nucleotides denoted in Figs 1 and 2. A stop codon mutation (UAA) in ORF2 of pCrPV-2 was introduced by mutating nucleotide A6428T of ORF2^34^. The numbering of the nucleotides is based on CrPV-2 in this case. Note that the only difference between CrPV-2 and CrPV-3 is the presence of a 196-nt duplication in the 5′UTR that does not affect IGR IRES translation. All plasmids were fully verified by sequencing to ensure no other mutations were in place.

### *In vitro* transcription and RNA transfection

Purified pCrPV-3 and derivative plasmids were linearized with Ecl136II. RNA was transcribed in a T7 RNA polymerase reaction and subsequently purified with a RNeasy kit (Qiagen). The integrity and purity of the RNA was confirmed on a 1.2% denaturing formaldehyde agarose gel.

Transfection of *in vitro* synthesized RNA into S2 cells was performed using Lipofectamine 2000 (Invitrogen) as per the manufacturer’s instructions. 3 μg of RNA derived from either pCrPV-3 or its cognate mutants were used for transfection using 2.5 × 10^6^ cells.

### Northern blot analysis

Total RNA was isolated from cells using TRIzol reagent. Northern blots were performed by loading 5 μg of RNA on a denaturing agarose gel and subsequently transferred to Zeta-probe blotting membrane (Bio-Rad). DNA probes were radiolabelled with a DecaLabel DNA labeling kit (Fermentas) and hybridized overnight. Radioactive bands were detected via phosphoimager analysis (Storm; GE Healthcare).

### Western blots

Equal amounts of S2 protein lysates (20 μg) were resolved on a 12% SDS-PAGE gel and then transferred to a polyvinylidene difluoride Immobilon-FL membrane (Millipore). Membranes were blocked for 30 min at room temperature with 5% skim milk in TBST. Blots were incubated for 1 h at room temperature with the following antibodies: CrPV ORF1 (raised against CrPV RdRp) rabbit polyclonal (1:10,000) or CrPV ORF2 (raised against CrPV VP2) rabbit polyclonal (1:10,000)^40^. Membranes were washed 3 times with TBST and incubated with either IRDye 800CW goat anti-rabbit IgG (1:20,000; LI-COR Biosciences) for 1 h at room temperature. An Odyssey imager (LI-COR Biosciences) was used for detection.

### *In vitro* and *in vivo* translation assays

*In vitro* translation of the full-length viral RNA genome (uncapped) was performed in *Spodoptera frugiperda* 21 (Sf-21) cell extract (Promega) in the presence of [^35^S]-methionine/cysteine. Reactions were loaded on a SDS-PAGE. Gels were dried and radioactive bands were monitored by phosphoimager analysis.

For *in vivo* translation assays, mock- or CrPV-infected S2 cells (MOI 5) were incubated with [^35^S]-methionine/cysteine for the last 30 minutes of the infection. Equal amounts of lysate (10 μg) were then loaded on a SDS-PAGE. Gels were dried and radioactive bands were monitored by phosphoimager analysis. Gels were quantified using ImageQuant software (GE Healthcare).

## Additional Information

**How to cite this article**: Kerr, C. H. *et al.* Molecular analysis of the factorless internal ribosome entry site in *Cricket Paralysis virus* infection. *Sci. Rep.* **6**, 37319; doi: 10.1038/srep37319 (2016).

**Publisher’s note**: Springer Nature remains neutral with regard to jurisdictional claims in published maps and institutional affiliations.

## Supplementary Material

Supplementary Information

## References

[b1] Jackson R. J., Hellen C. U. & Pestova T. V. The mechanism of eukaryotic translation initiation and principles of its regulation. Nature reviews. Molecular cell biology 11, 113–127 (2010). 20094052 10.1038/nrm2838 PMC4461372

[b2] Plank T. D. & Kieft J. S. The structures of nonprotein-coding RNAs that drive internal ribosome entry site function. Wiley interdisciplinary reviews. RNA 3, 195–212 (2012). 22215521 10.1002/wrna.1105 PMC3973487

[b3] Au H. H. & Jan E. Novel viral translation strategies. Wiley interdisciplinary reviews. RNA 5, 779–801 (2014). 25045163 10.1002/wrna.1246 PMC7169809

[b4] Pestova T. V., Shatsky I. N., Fletcher S. P., Jackson R. J. & Hellen C. U. A prokaryotic-like mode of cytoplasmic eukaryotic ribosome binding to the initiation codon during internal translation initiation of hepatitis C and classical swine fever virus RNAs. Genes Dev 12, 67–83 (1998). 9420332 10.1101/gad.12.1.67 PMC316404

[b5] Sweeney T. R., Abaeva I. S., Pestova T. V. & Hellen C. U. The mechanism of translation initiation on Type 1 picornavirus IRESs. EMBO J 33, 76–92 (2014). 24357634 10.1002/embj.201386124 PMC3990684

[b6] Bonning B. C. & Miller W. A. Dicistroviruses. Annu Rev Entomol 55, 129–150 (2010). 19961327 10.1146/annurev-ento-112408-085457

[b7] Khong A. *et al.* Temporal Regulation of Distinct Internal Ribosome Entry Sites of the Dicistroviridae Cricket Paralysis Virus. Viruses 8 (2016). 10.3390/v8010025 PMC4728584 26797630

[b8] Wilson J. E., Powell M. J., Hoover S. E. & Sarnow P. Naturally occurring dicistronic cricket paralysis virus RNA is regulated by two internal ribosome entry sites. Mol Cell Biol 20, 4990–4999 (2000). 10866656 10.1128/mcb.20.14.4990-4999.2000 PMC85949

[b9] Wilson J. E., Pestova T. V., Hellen C. U. & Sarnow P. Initiation of protein synthesis from the A site of the ribosome. Cell 102, 511–520 (2000). 10966112 10.1016/s0092-8674(00)00055-6

[b10] Sasaki J. & Nakashima N. Methionine-independent initiation of translation in the capsid protein of an insect RNA virus. Proc Natl Acad Sci USA 97, 1512–1515 (2000). 10660678 10.1073/pnas.010426997 PMC26465

[b11] Kanamori Y. & Nakashima N. A tertiary structure model of the internal ribosome entry site (IRES) for methionine-independent initiation of translation. RNA 7, 266–274 (2001). 11233983 10.1017/s1355838201001741 PMC1370084

[b12] Pfingsten J. S., Costantino D. A. & Kieft J. S. Structural Basis for Ribosome Recruitment and Manipulation by a Viral IRES RNA. Science 314, 1450–1454 (2006). 17124290 10.1126/science.1133281 PMC2669756

[b13] Fernandez I. S., Bai X. C., Murshudov G., Scheres S. H. & Ramakrishnan V. Initiation of Translation by Cricket Paralysis Virus IRES Requires Its Translocation in the Ribosome. Cell 8, 157, 823–831 (2014). 10.1016/j.cell.2014.04.015 PMC4017093 24792965

[b14] Muhs M. *et al.* Structural basis for the binding of IRES RNAs to the head of the ribosomal 40S subunit. Nucleic Acids Res 39, 5264–5275 (2011). 21378123 10.1093/nar/gkr114 PMC3130280

[b15] Costantino D. A., Pfingsten J. S., Rambo R. P. & Kieft J. S. tRNA-mRNA mimicry drives translation initiation from a viral IRES. Nat Struct Mol Biol 15, 57–64 (2008). 18157151 10.1038/nsmb1351 PMC2748805

[b16] Schuler M. *et al.* Structure of the ribosome-bound cricket paralysis virus IRES RNA. Nat Struct Mol Biol 13, 1092–1096 (2006). 17115051 10.1038/nsmb1177

[b17] Murray J. *et al.* Structural characterization of ribosome recruitment and translocation by type IV IRES. eLife 5, pii: e13567. doi: 10.7554/eLife.13567 (2016). PMC4861600 27159451

[b18] Jan E. & Sarnow P. Factorless ribosome assembly on the internal ribosome entry site of cricket paralysis virus. J Mol Biol 324, 889–902 (2002). 12470947 10.1016/s0022-2836(02)01099-9

[b19] Muhs M. *et al.* Cryo-EM of ribosomal 80S complexes with termination factors reveals the translocated cricket paralysis virus IRES. Mol Cell 57, 422–432 (2015). 25601755 10.1016/j.molcel.2014.12.016 PMC4526138

[b20] Spahn C. M. *et al.* Cryo-EM visualization of a viral internal ribosome entry site bound to human ribosomes: the IRES functions as an RNA-based translation factor. Cell 118, 465–475 (2004). 15315759 10.1016/j.cell.2004.08.001

[b21] Jan E., Kinzy T. G. & Sarnow P. Divergent tRNA-like element supports initiation, elongation, and termination of protein biosynthesis. Proc Natl Acad Sci USA 100, 15410–15415 (2003). 14673072 10.1073/pnas.2535183100 PMC307581

[b22] Pestova T. V. & Hellen C. U. Translation elongation after assembly of ribosomes on the Cricket paralysis virus internal ribosomal entry site without initiation factors or initiator tRNA. Genes Dev 17, 181–186 (2003). 12533507 10.1101/gad.1040803 PMC195975

[b23] Petrov A., Grosely R., Chen J., O’Leary S. E. & Puglisi J. D. Multiple Parallel Pathways of Translation Initiation on the CrPV IRES. Mol Cell 62, 92–103 (2016). 27058789 10.1016/j.molcel.2016.03.020 PMC4826567

[b24] Zhang H., Ng M. Y., Chen Y. & Cooperman B. S. Kinetics of initiating polypeptide elongation in an IRES-dependent system. eLife 5 (2016). 10.7554/eLife.13429 PMC4963199 27253065

[b25] Abeyrathne P. D., Koh C. S., Grant T., Grigorieff N. & Korostelev A. A. Ensemble cryo-EM uncovers inchworm-like translocation of a viral IRES through the ribosome. eLife 5, pii: e14874. doi: 10.7554/eLife.14874 (2016). PMC4896748 27159452

[b26] Zhu J. *et al.* Crystal structures of complexes containing domains from two viral internal ribosome entry site (IRES) RNAs bound to the 70S ribosome. Proc Natl Acad Sci USA 108, 1839–1844 (2011). 21245352 10.1073/pnas.1018582108 PMC3033271

[b27] Kamoshita N., Nomoto A. & RajBhandary U. L. Translation initiation from the ribosomal A site or the P site, dependent on the conformation of RNA pseudoknot I in dicistrovirus RNAs. Mol Cell 35, 181–190 (2009). 19647515 10.1016/j.molcel.2009.05.024 PMC2720879

[b28] Nishiyama T., Yamamoto H., Uchiumi T. & Nakashima N. Eukaryotic ribosomal protein RPS25 interacts with the conserved loop region in a dicistroviral intergenic internal ribosome entry site. Nucleic Acids Res 35, 1514–1521 (2007). 17287295 10.1093/nar/gkl1121 PMC1865070

[b29] Landry D. M., Hertz M. I. & Thompson S. R. RPS25 is essential for translation initiation by the Dicistroviridae and hepatitis C viral IRESs. Genes Dev 23, 2753–2764 (2009). 19952110 10.1101/gad.1832209 PMC2788332

[b30] Jang C. J., Lo M. C. & Jan E. Conserved element of the dicistrovirus IGR IRES that mimics an E-site tRNA/ribosome interaction mediates multiple functions. J Mol Biol 387, 42–58 (2009). 19361441 10.1016/j.jmb.2009.01.042

[b31] Ruehle M. D. *et al.* A dynamic RNA loop in an IRES affects multiple steps of elongation factor-mediated translation initiation. eLife 4, pii: e08146. doi: 10.7554/eLife.08146 (2015). PMC4709265 26523395

[b32] Au H. H. & Jan E. Insights into factorless translational initiation by the tRNA-like pseudoknot domain of a viral IRES. PLoS One 7, e51477 (2012). 23236506 10.1371/journal.pone.0051477 PMC3517527

[b33] Jack K. *et al.* rRNA pseudouridylation defects affect ribosomal ligand binding and translational fidelity from yeast to human cells. Mol Cell 44, 660–666 (2011). 22099312 10.1016/j.molcel.2011.09.017 PMC3222873

[b34] Kerr C. H. *et al.* The 5′ untranslated region of a novel infectious molecular clone of the dicistrovirus cricket paralysis virus modulates infection. J Virol 89, 5919–5934 (2015). 25810541 10.1128/JVI.00463-15 PMC4442438

[b35] Au H. H. *et al.* Global shape mimicry of tRNA within a viral internal ribosome entry site mediates translational reading frame selection. Proc Natl Acad Sci USA 112, E6446–E6455 (2015). 26554019 10.1073/pnas.1512088112 PMC4664324

[b36] Jang C. J. & Jan E. Modular domains of the Dicistroviridae intergenic internal ribosome entry site. RNA 16, 1182–1195 (2010). 20423979 10.1261/rna.2044610 PMC2874170

[b37] Thompson S. R., Gulyas K. D. & Sarnow P. Internal initiation in Saccharomyces cerevisiae mediated by an initiator tRNA/eIF2-independent internal ribosome entry site element. Proc Natl Acad Sci USA 98, 12972–12977 (2001). 11687653 10.1073/pnas.241286698 PMC60809

[b38] Fernandez J., Yaman I., Sarnow P., Snider M. D. & Hatzoglou M. Regulation of internal ribosomal entry site-mediated translation by phosphorylation of the translation initiation factor eIF2alpha. J Biol Chem 277, 19198–19205 (2002). 11877448 10.1074/jbc.M201052200

[b39] Christian P. D. & Scotti P. D. Picornalike viruses of insects, In The insect viruses 301–336 (Springer, 1998).

[b40] Garrey J. L., Lee Y. Y., Au H. H., Bushell M. & Jan E. Host and viral translational mechanisms during cricket paralysis virus infection. J Virol 84, 1124–1138 (2010). 19889774 10.1128/JVI.02006-09 PMC2798387

[b41] Krishna N. K., Marshall D. & Schneemann A. Analysis of RNA packaging in wild-type and mosaic protein capsids of flock house virus using recombinant baculovirus vectors. Virology 305, 10–24 (2003). 12504536 10.1006/viro.2002.1740

