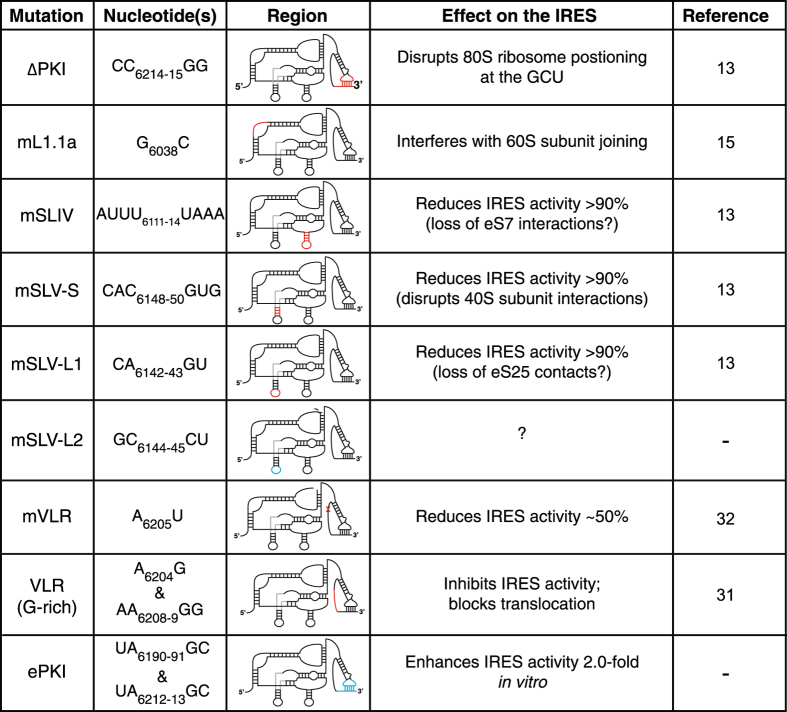# Erratum: Molecular analysis of the factorless internal ribosome entry site in *Cricket Paralysis virus* infection

**DOI:** 10.1038/srep39439

**Published:** 2017-04-04

**Authors:** Craig H. Kerr, Zi Wang Ma, Christopher J. Jang, Sunnie R. Thompson, Eric Jan

Scientific Reports 6: Article number: 3731910.1038/srep37319; published online: 11 17 2016; updated: 04 04 2017

This Article contains an error in Figure 2 in the column labelled ‘Nucleotide(s)’, row ‘mSLV-L2’. The subscript text ‘6144–45’ is missing between GC and CU. In addition, there is an error in the mutation column where mVLR is incorrectly given as VLRm. The correct Figure 2 appears below as Figure 1.

## Figures and Tables

**Figure 1:**