# Supplementary material for: Molecular analysis of the factorless internal ribosome entry site in Cricket Paralysis virus infection
Source: Sci Rep. 2016 Nov 17;6:37319. doi: 10.1038/srep37319 (PMC5112510; doi:10.1038/srep37319)

Molecular analysis of the factorless internal ribosome entry site in *Cricket Paralysis virus* infection

Craig H. Kerr[1], Zi Wang Ma[1], Christopher J. Jang[1], Sunnie R. Thompson[2] and Eric Jan[1]*

[1]Department of Biochemistry and Molecular Biology, University of British Columbia, Vancouver BC, Canada and [2]Department of Microbiology, University of Alabama at Birmingham, Birmingham, Alabama, USA

*corresponding author: ej@mail.ubc.ca

SUPPLEMENTARY FIGURE LEGENDS

Supplemental Figure 1. Uncropped gel images of those seen in Figure 2.

Supplemental Figure 2. Uncropped images of those seen in Figure 3.

Supplemental Figure 3. Uncropped images of those seen in Figure 4.

Supplemental Figure 1 Kerr *et al.*

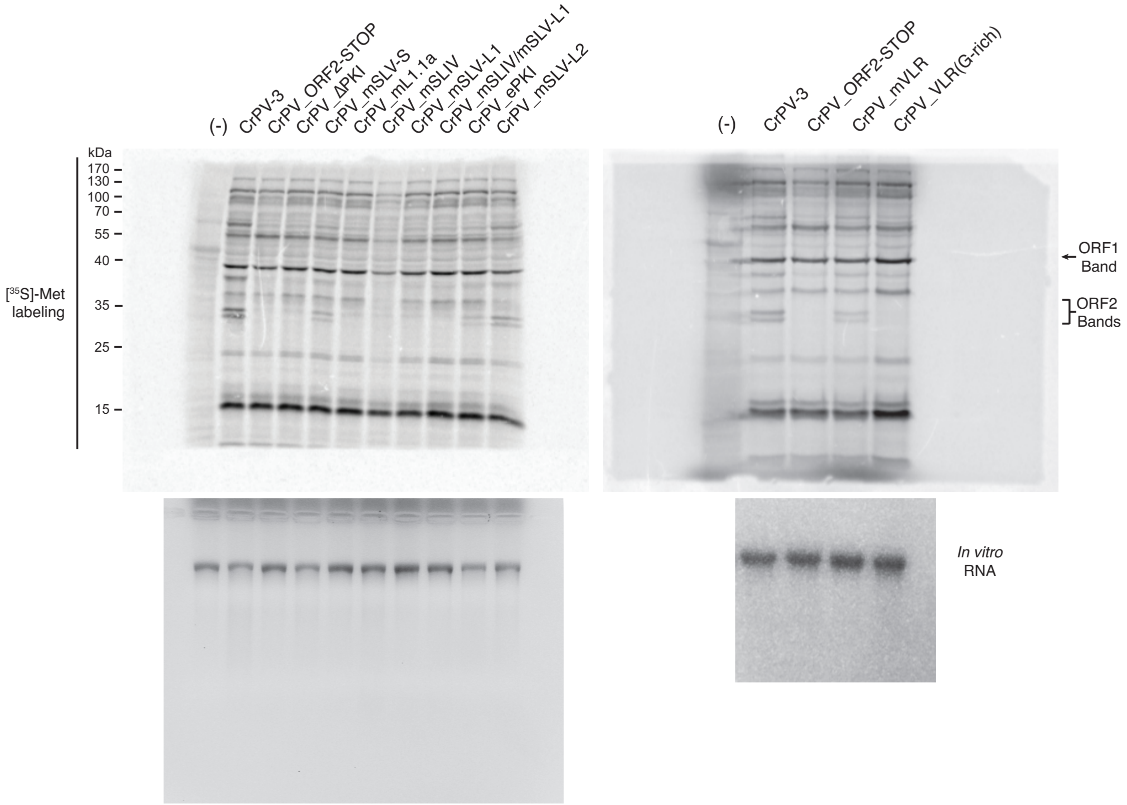

Supplemental Figure 2 Kerr *et al.*

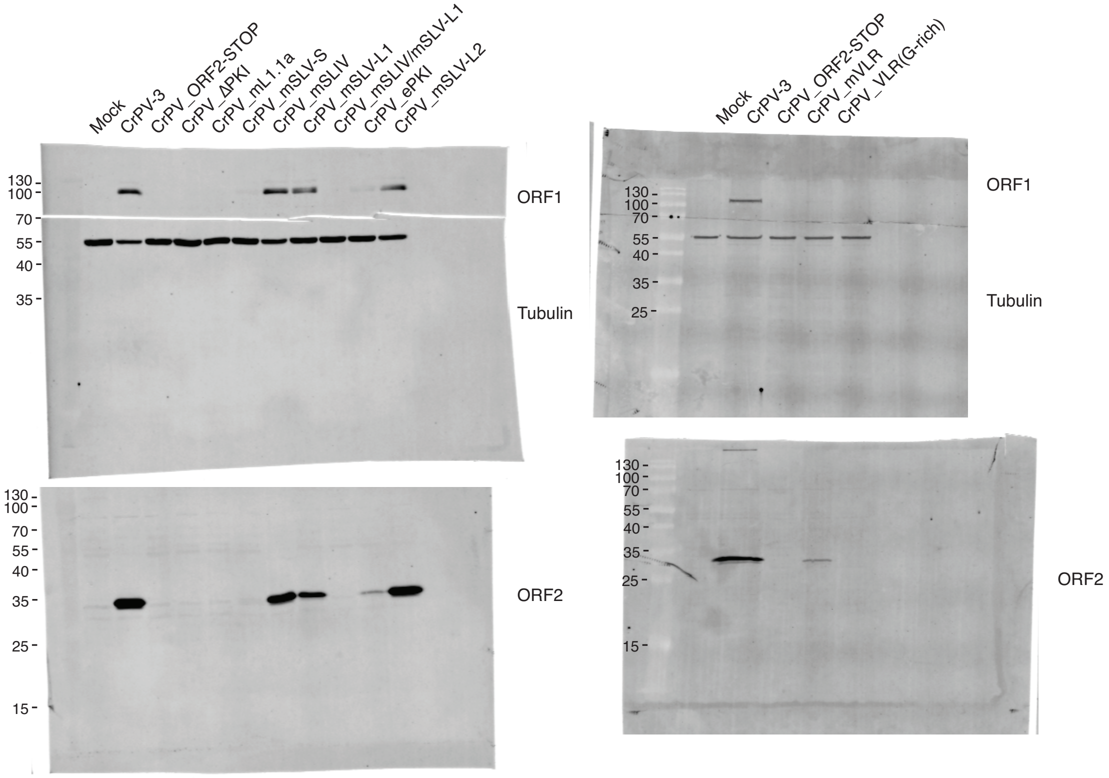

Supplemental Figure 3 Kerr *et al.*

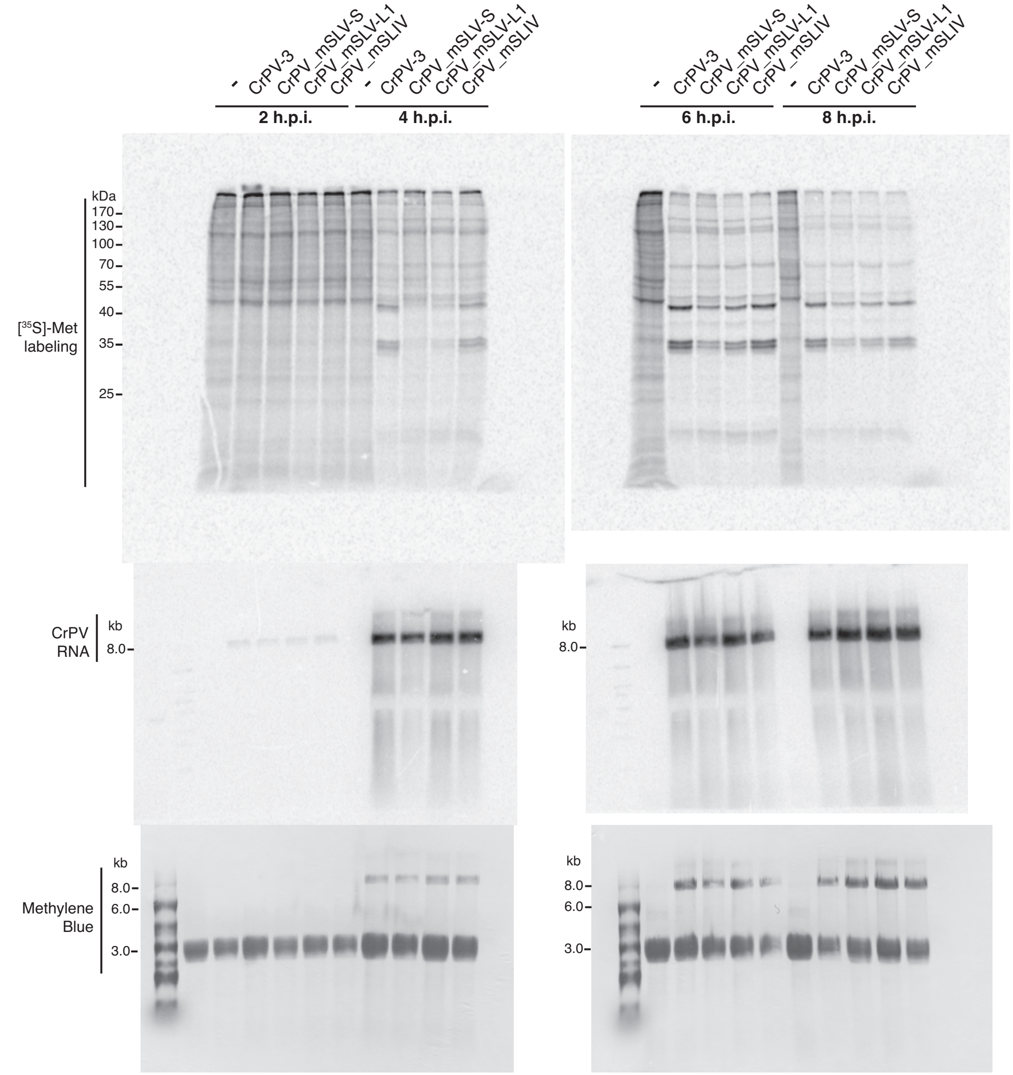

Supplement: Supplementary Information [file srep37319-s1.pdf]
